# Extreme multiexciton emission from deterministically assembled single-emitter subwavelength plasmonic patch antennas

**DOI:** 10.1038/s41377-020-0269-0

**Published:** 2020-03-04

**Authors:** Amit Raj Dhawan, Cherif Belacel, Juan Uriel Esparza-Villa, Michel Nasilowski, Zhiming Wang, Catherine Schwob, Jean-Paul Hugonin, Laurent Coolen, Benoît Dubertret, Pascale Senellart, Agnès Maître

**Affiliations:** 1 Institute of Fundamental and Frontier Sciences, University of Electronic Science and Technology of China, Chengdu, 610054 People’s Republic of China; 2 Sorbonne Université, CNRS, Institut des Nanosciences de Paris, UMR 7588, 75005 Paris, France; 3 Centre de Nanosciences et de Nanotechnologies et de Nanostructures, CNRS UMR9001, Université Paris-Saclay, 10 boulevard Thomas Gobert, 91120 Marcoussis, France; 4 Laboratoire de Physique et d’Etude des Matériaux, ESPCI-ParisTech, PSL Research University, Sorbonne Université, CNRS UMR 8213, 10 rue Vauquelin, Paris, 75005 France; 5 Laboratoire Charles Fabry, Institut d’Optique Graduate School, CNRS UMR 8501, Université Paris Saclay, 2 avenue Augustin Fresnel, 91127 Palaiseau Cedex, France

**Keywords:** Nanophotonics and plasmonics, Quantum dots

## Abstract

Coupling nano-emitters to plasmonic antennas is a key milestone for the development of nanoscale quantum light sources. One challenge, however, is the precise nanoscale positioning of the emitter in the structure. Here, we present a laser etching protocol that deterministically positions a single colloidal CdSe/CdS core/shell quantum dot emitter inside a subwavelength plasmonic patch antenna with three-dimensional nanoscale control. By exploiting the properties of metal–insulator–metal structures at the nanoscale, the fabricated single-emitter antenna exhibits a very high-Purcell factor (>72) and a brightness enhancement of a factor of 70. Due to the unprecedented quenching of Auger processes and the strong acceleration of the multiexciton emission, more than 4 photons per pulse can be emitted by a single quantum dot, thus increasing the device yield. Our technology can be applied to a wide range of photonic nanostructures and emitters, paving the way for scalable and reliable fabrication of ultra-compact light sources.

## Plasmonic antennas: precise alignment for high brightness

Finding practical ways to control and enhance the emission from single-photon sources is important for applications in quantum optics spanning from fundamental science to quantum information processing. While plasmonic antennas are a solution, the accurate alignment and coupling of such antennas to single-photon emitters like quantum dots, vacancy-centrenanodiamonds and fluorescent molecules is extremely challenging. Laser etching can help ease the task, allowing a CdSe/CdS core-shell quantum dot to be deterministically placed inside a subwavelength plasmonic patch antenna with 3 nm vertical and 50 m lateral precision. Amit Raj Dhawan and coworkers from Chengdu, China and Paris, France, show that the precise control over the location of the emitter results in an emitter-antenna system with a brightness enhancement of a factor of 70 and a large Purcell factor >72.

## Introduction

The interaction between an emitter and its local electromagnetic field can be engineered by increasing the local density of states for applications in quantum information^1^ and single-photon generation^2^. This approach has been widely explored with a large variety of dielectric^3–5^ or plasmonic environments^6–10^ and solid-state emitters such as self-assembled^11^ or colloidal^12^ quantum dots (QDs), single molecules^13^, and defects in diamond^14^. Achieving an intense light–matter interaction using extremely small mode volume plasmonic structures^12,15^ requires very precise, and hence challenging, spatial positioning of the emitter at the nanoscale. A large majority of the works so far have relied on randomly positioned emitters to demonstrate strong acceleration of the spontaneous emission of single emitters coupled to plasmonic antennas^16–18^. The challenge of deterministically positioning individual emitters inside nanophotonic structures with three-dimensional nanoscale control has to be overcome to realize efficient devices operating at room temperature and benefiting from highly optimized light–matter interaction regimes^16,19^.

Several approaches have been developed to precisely position single self-assembled quantum dots grown by molecular beam-epitaxy in cavities^20–23^. These approaches rely on optically measuring the QD position through emission mapping with subwavelength accuracy and defining the photonic structure around the QD using optical^20–22^ or electronic^23^ lithography. However, these techniques cannot be extended to chemically synthetized emitters such as colloidal QDs or molecules that have strong potential for inexpensive and room-temperature applications. The main reason for this limitation is the exacerbated sensitivity of these emitters to technological processing. Because of the high sensitivity of the emission process to surface states, preserving the properties of the colloidal QDs during technological protocols, involving, for example, e-beam or laser exposure, dry or wet etchings and solvents, is highly challenging.

Here, we report a non-destructive in situ far-field laser etching lithography technique that allows control—at the nanoscale—of the position of fragile emitters in plasmonic structures. Our technique employs multilayer structures and a dual-wavelength protocol to deterministically position a single CdSe/CdS colloidal QD in a plasmonic patch antenna and fabricate subwavelength antennas of different shapes. As a result, we deterministically achieve a high Purcell factor for a single emitter. Strong electromagnetic interactions in the plasmonic antenna decrease the relative efficiency of the Auger recombination channels, which results in very high brightness and extreme multiexciton radiative recombination. The technique can be used to fabricate a variety of photonic structures embedding fragile emitters.

## Results

### Plasmonic patch antenna for spontaneous emission control

Optimizing the coupling between an emitter and a nanostructure makes it possible to control the emission directivity and the dynamics of the spontaneous emission. The latter is quantified by the Purcell factor *F*_P_, which scales as the inverse of the electromagnetic confinement volume provided by the photonic structure^4,24,25^. Plasmonic nanoantennas^6^ exhibit a very low mode volume and a wide spectral resonance and therefore are excellent structures for obtaining high Purcell factors *F*_P_ with broadband emitters.

**Fig. 1:**
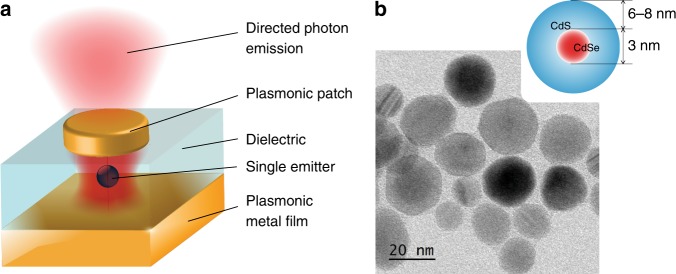
A plasmonic patch antenna coupled to a single colloidal quantum dot. **a** Schematic of the structure under study consisting of a single colloidal core/shell QD coupled to a plasmonic patch antenna. **b** Schematic and transmission electron microscopy image of the investigated emitters

Figure 1a depicts the system that is explored in the present work: a single emitter coupled to a plasmonic patch antenna. The antenna consists of a thin dielectric layer (typically 30–40 nm) sandwiched between an optically thick bottom layer of gold and a thin gold patch on top; the patch thickness is on the order of 20 nm, and its diameter is in the range of 0.2–2.5 μm. This system has been both theoretically and experimentally shown to be an excellent tool for accelerating and directing fluorescence emission^26,27^.

The optimal positioning of the emitter inside the antenna couples the radiation of the emitter to surface plasmon polaritons (SPPs) at both nano-spaced metal-dielectric interfaces, and the SPPs in the patch are confined by the geometry of the patch. These SPPs at the two interfaces further couple and create strong confinement of the electromagnetic field around the emitter. The SPPs generated in the thin plasmonic metal patch (thinner than the skin depth) lead to the emission of photons^26^ as depicted in Fig. 1a. The antenna operation depends on the dipole orientation of the emitter (stronger acceleration of the spontaneous emission for the vertical dipole orientation), the patch size (large patches are more directive, but small patches show stronger resonances), and the dielectric spacer^26^. In this work, we insert chemically synthetized^28^ relatively large quasi-spherical individual CdSe/CdS semiconductor core/shell colloidal QDs^29,30^ in the antenna. These QDs have CdSe cores that are ~3 nm in diameter and encapsulated by slowly grown CdS shells with a thickness of 6–8 nm (Fig. 1b), which make them almost non-blinking^31^. Due to their high absorption cross-section, these QDs exhibit bright fluorescence at room temperature. Under ultra-violet (UV) excitation at room temperature, they emit at 633 nm with a spectral width of 30 nm. We characterize these QDs in the low-excitation limit, where Auger processes are efficient enough to lead to single-photon emission. We note that under strong pumping, these large single QDs emit multiple photons because their multiexciton emission rate becomes comparable to the non-radiative Auger rate (see Supplementary Information).

### In situ subwavelength laser etching on fragile emitters

**Fig. 2:**
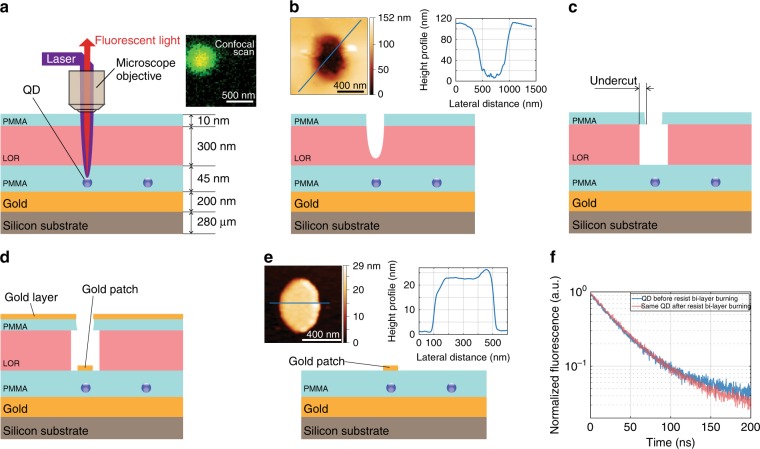
In situ laser etching for the deterministic assembly of single-emitter plasmonic antenna nanostructures. Figures (**a**–**e**) illustrate the in situ laser etching protocol used to fabricate the antennas (see the details in the text). The image at the top right of (**a**) shows a confocal image of the emission from a QD, and the image on the top of (**b**) shows the AFM topography and height profile of a hole etched by the laser into the resist bi-layer. The undercut created by the selective etching of the LOR (**c**) allows the lift-off in step (**d**) of the lithography to produce the single-emitter antennas (**e**). The image above (**e**) depicts the AFM image and corresponding height profile of a fabricated antenna patch. **f** Emission lifetime of a QD before (blue) and after (red) laser etching

The proposed technological protocol is illustrated in Fig. 2 and consists of deterministic and non-destructive in situ laser etching, which allows the positioning of a single QD within an antenna with a 3 nm vertical precision and a 50 nm lateral precision (see Supplementary Information). We use a low-luminescence bi-layer polymer^32^ to locate single emitters by mapping their luminescence.

The process starts by evaporating a thick layer of gold (200 nm) on a Si substrate using an intermediate adhesion layer of Ti/Cr. A 10-nm thick layer of polymethyl methacrylate (PMMA) is then spin-coated above gold. Then, a layer of spatially well-isolated single QDs is spin-coated using a dispersion of QDs in hexane of an appropriate dilution. To create an optimized spacing around the single emitters and to protect them from direct dielectric vapour deposition, a 35-nm thick smooth PMMA film is spin-coated, which embeds the emitters in a dielectric layer. This protects the emitters in the proceeding lithographic steps. A bi-layer consisting of lift-off resist (LOR) and PMMA is then spin-coated. The low luminosity of this bi-layer permits the detection of the emission from the single QDs embedded beneath and excited by a low-intensity spectrally filtered broadband supercontinuum laser emitting at 473–478 nm (Fig. 2a). As in the case of in situ lithography methods^20,23^, we map the QD fluorescence with nanoscale accuracy. The laser wavelength is then tuned to 550–605 nm, which corresponds to a wavelength range where the laser light is absorbed substantially more by the lift-off resist than by the QD. The excitation power can thus be chosen to burn the resist bi-layer and locally create a hole directly above the selected QD without photobleaching the QD (Fig. 2b). We note in Fig. 2f, the emission lifetime of a QD does not change after the resist burns—this demonstrates that the laser etching process does not photodegrade the QD. After selective chemical etching of the LOR, an undercut is created in the PMMA (Fig. 2c). Finally, gold is deposited by evaporation (Fig. 2d), and a lift-off step is performed to obtain patch antennas embedding a single emitter (Fig. 2e). The undercut in the PMMA leads to a discontinuous deposition of gold (thus making the patch), and in step (e), the undercut provides a passage for the chemical etching agent to attack the LOR and lift-off the layers above the LOR while leaving the patch. The atomic force microscopy (AFM) image above Fig. 2e shows an antenna patch after the final lift-off step.

**Fig. 3:**
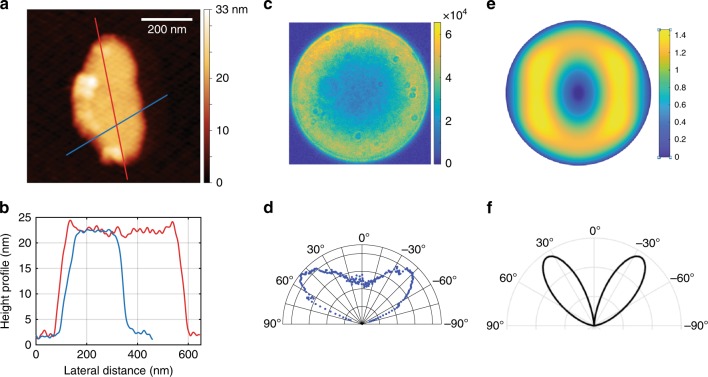
Topography and emission pattern of the emitter-antenna structures. **a** AFM image of a small patch antenna and **b** the corresponding height profiles. **c** Electron-multiplying charge-coupled device (EMCCD) camera image of the radiation pattern of the same antenna measured in the far field by imaging the Fourier plane of the QD emission and **d** the associated polar plot. **e** (*θ*, *φ*) polar plot of the numerically simulated emission pattern of a similar elliptical patch antenna and **f** the polar plot along the maximum intensity axis

This laser etching technique has several advantages over previous methods. Standard optical in situ lithography would require intense UV laser light for the exposure, which would degrade the QDs due to excessive light absorption. Here, by using a selected wavelength of 550–605 nm for the laser etching, we avoid such a detrimental effect. Moreover, this technique allows one to scan and locate the emitter without exposing the photoresist. Finally, as the etching process is highly nonlinear with respect to the excitation power, subwavelength structures can be obtained. The AFM image and height profile of the antenna patch in Fig. 3a, b, respectively, show a nano-antenna with dimensions as small as 200 × 450 nm^2^, while the mean wavelength of the etching laser is 580 nm. As the technique is deterministic, it is possible to characterize the emitter (in terms of the emission decay, polarization response, photon correlation, and spectral response) before it is embedded in the antenna. By noting antibunching in the QD emission before the in situ lithography, it is assured that the subsequent antenna will contain only one QD. The power, wavelength and exposure time of the laser can be optimized to prevent the degradation of the QDs after a successful lithography process. For the QD batch discussed in the paper, we noted that more than 80% of the QDs survived the laser etching process, from which all of the QDs survived the patch deposition process. This protocol ensures that all the fabricated antennas are single-emitter antennas.

### Emission from a subwavelength antenna

We study the emission pattern of the very small antenna (Fig. 3a, b) coupled to a single QD. Such subwavelength plasmonic patch antennas have been predicted to show a very strong Purcell effect and directive emission^7,26^. Simulation studies have shown that these antennas exhibit a higher *F*_P_ if the emitter dipole is perpendicular rather than parallel to the patch. Moreover, for a perpendicular dipole, by reducing the patch diameter and employing specific patch sizes, *F*_P_ can increase from 70 (patch diameter of ~1.6 μm) to more than 160 (patch diameter of ~0.15 μm). Therefore, the ability to reduce the antenna patch to a subwavelength size is a tool that can be used to control the emission acceleration.

Figure 3c, d shows the radiation pattern of the antenna measured in the far field by Fourier plane imaging. The antenna radiates through a cone in the far field, centred around 45°. This measurement is in good agreement with the numerical simulation of an elliptical patch antenna using the Fourier modal method^33–36^ as depicted in Fig. 3e, f for the resonance wavelength at 604 nm.

A striking feature of the measured radiative pattern is its high symmetry in the far field. Indeed, if the emitter is slightly off-centred with respect to the antenna patch, the radiation pattern rapidly exhibits angular asymmetry^7^. The lobes of Fig. 3d, f demonstrate that the emitter is centred with respect to the antenna within 5 nm, thus exhibiting the precision of the fabrication method used to position the QD laterally at the centre of the patch.

### Acceleration of spontaneous emission

**Fig. 4:**
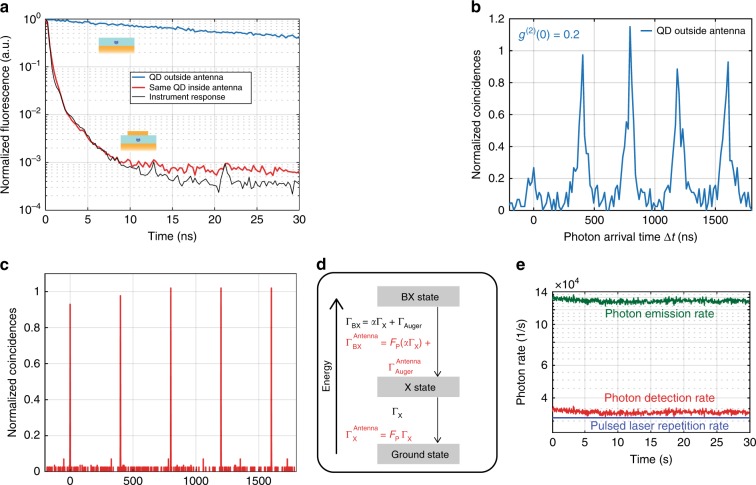
Emission characteristics of a highly accelerated antenna. **a** Fluorescence decay of the QD before (blue line) and after (red line) it is placed inside the antenna. The instrument response function is shown in black. **b** Normalized photon coincidences measured for the QD before insertion in the antenna and **c** inside the antenna under a pulsed excitation of 0.03 W/cm^2^ at 405 nm. **d** QD energy-level diagram showing the biexciton (BX), exciton (X), and ground states. **e** Detected photon rate (red) and the corresponding photon emission rate after the detection efficiency correction (green). The blue line denotes the pulsed laser repetition rate

Figure 4a displays the fluorescence decay of a given QD before and after it is placed inside the patch antenna of Fig. 3. Before the deposition of the upper gold patch, when the QD is embedded in the PMMA layer and is 10 nm above the gold surface, its emission decay is monoexponential in character, which is indicative of single-exciton recombination with a decay of $\tau _{\mathrm{X}}^{{\mathrm{ref}}}$ = 36 ns. After the deposition of a gold patch centred on this single QD, the emission lifetime is considerably reduced from $\tau _{\mathrm{X}}^{{\mathrm{ref}}}$ to $\tau _{\mathrm{X}}^{{\mathrm{Antenna}}}$, and the acceleration of the spontaneous emission $F_{\mathrm{P}}^{{\mathrm{Antenna}}} = \frac{{\tau _{\mathrm{X}}^{{\mathrm{Antenna}}}}}{{\tau _{\mathrm{X}}^{{\mathrm{ref}}}}}$ of a given single QD can be measured. As the proximity to gold shortens the emitter lifetime^37–40^, we estimate that, on average, the lifetime $\tau _{\mathrm{X}}^{{\mathrm{ref}}}$ of a single QD just above the gold (and not inside the antenna) is three times shorter than the decay time of the same colloidal QDs in a homogeneous PMMA layer, i.e., $\frac{{\tau _{\mathrm{X}}^{{\mathrm{homogeneous}}}}}{{\tau _{\mathrm{X}}^{{\mathrm{ref}}}}}$ = 3 (see Supplementary Information), where $\tau _{\mathrm{X}}^{{\mathrm{homogeneous}}}$ is the exciton lifetime of the emitter in a homogeneous medium (infinite dielectric medium with a refractive index of 1.5, such as PMMA). The Purcell factor is calculated using the exciton decay rate and is given as $F_{\mathrm{P}} = \frac{{\tau _{\mathrm{X}}^{{\mathrm{homogeneous}}}}}{{\tau _{\mathrm{X}}^{{\mathrm{ref}}}}} \times \frac{{\tau _{\mathrm{X}}^{{\mathrm{ref}}}}}{{\tau _{\mathrm{X}}^{{\mathrm{Antenna}}}}}$.

Note that the acceleration of the spontaneous emission observed in Fig. 4a is so strong that we are limited by the response time of the measurement instrument to precisely quantify *F*_P_. By analysing the instrument response function (see Supplementary Information), we find that the emission acceleration is >24 with respect to the lifetime of the same QD before the gold patch deposition, which results in *F*_P_ > 72 (detailed in Supplementary Information), when compared with the case of a QD embedded in a homogeneous dielectric medium. We remark that the stated value of *F*_P_ was found by comparing the exciton lifetime of the QD with the slowest component of the instrument response function and not the faster component, resulting in a lower but more accurate value of *F*_P_. The method is detailed and justified in the Supplementary Information. In comparison to previous studies^7,12,41^, this extreme Purcell effect for a single emitter constitutes a high value that is obtained by the controlled and deterministic positioning of the emitter at the nanoscale in a highly confining subwavelength antenna structure.

### Quenching of the Auger processes and multiexciton emission

As a consequence of such a significant Purcell effect, the electromagnetic decay channels are accelerated, making the radiative multiexciton recombination more efficient than the Auger non-radiative channels. The photon-correlation curve of Fig. 4b shows the measured second-order intensity correlation of the QD emission before it is placed in the antenna, exhibiting single-photon emission with *g*^(2)^(0) = 0.2 (calculated using a 15 ns binning interval). When inserted in the antenna, this value increases to *g*^(2)^(0) = 1 (Fig. 4c, calculated using a 0.5 ns binning interval), confirming the radiative multiexciton recombination.

Inside the plasmonic antenna, the radiative decay rates are accelerated by the Purcell factor *F*_P_ not only at the exciton level but also at the multiexciton level. As a result, the Auger processes that are very efficient at the multiexciton level become relatively inefficient in the high-Purcell-factor regime, which facilitates the radiative recombination of the multiexcitons^42–44^. This is substantiated by the loss of single-photon emission in Fig. 4c. The three-level system in Fig. 4d shows the different decay channels for a QD in a homogeneous medium (black colour) and the QD in an environment with a Purcell effect (red colour), such as a plasmonic antenna. In the homogeneous medium, the QD decays in a cascade from the biexciton (BX) to the exciton (X) state with a rate of ${\mathrm{\Gamma }}_{{\mathrm{BX}}} = \alpha {\mathrm{\Gamma }}_{\mathrm{X}} + {\mathrm{\Gamma }}_{{\mathrm{Auger}}}$ and from the exciton to the ground state with a rate of Γ_X_. When the same QD is placed in an environment such as a plasmonic antenna, due to the Purcell effect, the QD decays in a cascade from the biexciton to the exciton state with a rate of ${\mathrm{\Gamma }}_{{\mathrm{BX}}}^{{\mathrm{Antenna}}} = F_{\mathrm{P}}(\alpha {\mathrm{\Gamma }}_{\mathrm{X}}) + {\mathrm{\Gamma }}_{{\mathrm{Auger}}}^{{\mathrm{Antenna}}}$ and from the exciton to the ground state with a rate of *F*_P_Γ_X_. For the homogeneous and antenna media, Γ_BX_ and ${\mathrm{\Gamma }}_{{\mathrm{BX}}}^{{\mathrm{Antenna}}}$ are the biexciton decay rates, Γ_X_ and ${\mathrm{\Gamma }}_{\mathrm{X}}^{{\mathrm{Antenna}}}$ are the exciton decay rates, and Γ_Auger_ and ${\mathrm{\Gamma }}_{{\mathrm{Auger}}}^{{\mathrm{Antenna}}}$ are the Auger decay rates, respectively. The Purcell factor *F*_P_ accelerates all channels except the Auger channels. The factor *α* ≥ 2 relates the biexciton and exciton decay rates^45,46^. To calculate *F*_P_, we use the experimentally measured Γ_X_ and ${\mathrm{\Gamma }}_{\mathrm{X}}^{{\mathrm{Antenna}}}$ and avoid comparing Γ_BX_ and ${\mathrm{\Gamma }}_{{\mathrm{BX}}}^{{\mathrm{Antenna}}}$ to find *F*_P_ as this requires a separation of the Auger decay rates, which are not measured distinctively.

The plot of Fig. 4e illustrates very strong radiative multiexciton emission from a single-emitter plasmonic optical antenna. Under 405 nm pulsed excitation at a rate of 31.25 kHz with a pulse width of ~100 ps, the photon rate detected by the photodiodes is ~34 kHz. Considering the 25% detection efficiency of the experimental setup, the photon emission rate from the QD is estimated to be ~136 kHz. Therefore, for each laser pulse, the single emitter in the antenna emits ~4–5 photons. This demonstrates extremely efficient quenching of the Auger processes for at least 5 levels of radiative multiexciton recombination. Most likely, the relaxation of the QD inside the antenna includes many more higher multiexciton levels for which the non-radiative Auger recombination is not completely quenched, which therefore do not contribute to the radiation.

**Fig. 5:**
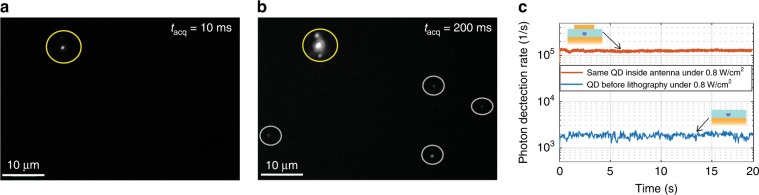
Fluorescence enhancement by a plasmonic antenna. **a**, **b** Fluorescence microscopy image of the antenna excited by a mercury lamp at 438 ± 12 nm and captured by a CCD camera with an acquisition time of 10 ms (**a**) or 200 ms (**b**). The fluorescing antenna is encircled in yellow. Note that the two lighter spots above and below the saturated antenna spot in (**b**) are reflections on the protecting glass before the CCD camera sensor. **c** Photon detection rate from the QD before (blue) and after (red) it is placed inside the antenna. The antenna is excited at 405 nm with a pulsed laser at 2.5 MHz

While a high Purcell effect in plasmonic antennas has often been associated with low brightness due to the predominance of non-radiative channels^47–50^, the structures developed here exhibit a very high fluorescence enhancement. Figure 5a, b are charge-coupled device (CCD) camera fluorescence images of the same area, including the small antenna of Fig. 3 and some QDs scattered outside the antenna. Under excitation with a lamp (438 ± 12 nm), with an acquisition time of 10 ms, only the antenna emission (indicated by the yellow circle in Fig. 5a) can be detected. An acquisition time of 200 ms (Fig. 5b) is required to detect the emission from the QDs outside antennas. To quantify the brightness of the antenna, we use a pulsed laser at 405 nm to excite the QD before and after insertion in the antenna. For the same excitation power, the antenna signal is 72 times more intense (Fig. 5c).

We also note the high optical quality and stability of the fabricated antenna. The antenna can sustain laser pulses with an instantaneous power of 120 mW for more than 10 min before it is photobleached—evidencing the quality of the QDs under study and their preservation during the laser etching protocol reported here. We finally note that the onset of multiexciton emission depends strongly on the antenna size and the Purcell effect due to the antenna. In general, the appearance of multiexciton emission depends on the relative weight of the radiative decay of the multiexciton states and the Auger rate. When increasing the number of excitons, the corresponding multiexciton emission rate increases. In the presence of efficient Auger processes, it is thus possible to observe multiexciton emission when increasing the excitation power to the extent that the multiexciton radiative decay rate exceeds the Auger rate. We have observed this effect studying several antennas of different Purcell factors. For larger (hence slower) antennas, the onset of multiexciton emission is typically observed at a larger excitation power than for smaller (hence faster) antennas. This effect can be used in applications that demand switching from single-photon emission to multiphoton emission^51^. Controlled room-temperature multiexciton radiation can benefit photonic technologies^52^ and improve the efficiency of light-emitting devices^53,54^.

## Discussion

We have developed a novel deterministic laser etching protocol that enables the fabrication of subwavelength plasmonic antennas centred with a 50 nm lateral accuracy on a single colloidal QD. Due to the low mode volume and high field confinement, such an antenna exhibits a remarkable Purcell effect, directive emission and high brightness.

Further developments of the protocol include the selection of well-oriented emitters by polarization analysis^49–51^ for fully deterministic matching of the spectrum, location and orientation between the emitter and the antenna modes for optimized light–matter interactions. To reduce the losses in such structures, where more than 90% of the plasmonic energy can be lost as heat^48^, the fabrication capabilities of the protocol can be extended to produce hybrid structures such as metallo-dielectric antennas.

The same protocol can be used to synthesize other nanophotonic devices, plasmonic waveguides^55^ and nanolasers^56,57^, which require deterministic and controlled positioning of a variety of sensitive emitters operating at room temperature, such as small colloidal QDs (e.g., CdSe/CdS and PbSe/PbS), nanodiamonds with vacancy centres such as nitrogen vacancies and silicon vacancies, and fluorescent molecules. Using this technique, while controlling the antenna shape and size and the dipolar orientation of the emitter inside the antenna and by using emitters with low multiexciton emission, very bright and directive single-photon sources based on such antennas can be obtained.

## Materials and methods

### Sample preparation

The CdSe cores of the colloidal CdSe/CdS core/shell QDs were synthesized using a protocol based on the work of Peng et al.^28^, and the CdS shells were grown using the continuous slow injection method. On a 0.28-mm thick polycrystalline Si substrate, a 200 nm layer of Au (refractive index of *n* = 0.0534 + *i*3.8249 at 630 nm after deposition measured by ellipsometry) was grown by thermal evaporation using an adhesion layer of Cr (10 nm). A 10-nm-thick film of polymethyl methacrylate (PMMA) was deposited on the Au substrate by spin-coating a solution of 0.5% [m/m] PMMA (average molar mass of 101,000) in toluene at 4000 rpm for 40 s. The sample was baked at 150 °C for 2 min. Then, a dispersion of CdSe/CdS QDs in hexane was spin-coated at 4000 rpm for 40 s to obtain well-distributed individual QDs. Then, by spin-coating 1.5% [m/m] of PMMA solution in toluene at 4000 rpm for 40 s on the QDs and baking the substrate at 150 °C for 2 min, individual QDs were embedded inside a dielectric layer of PMMA with *n* = 1.50 at 630 nm. To perform optical lithography on the sample, a lift-off resist (LOR®3A) was spin-coated at 7000 rpm for 40 s, and the sample was baked at 150 °C for 2 min, which produced a 300-nm thick layer. Then a 10-nm thick PMMA layer was deposited by spin-coating a solution of 0.5% [m/m] PMMA in toluene, and the sample was baked at 150 °C for 2 min.

### Lithography protocol

The protocol has been described in the paper.

### Optical characterization and microscopy

The sample was mounted on a PI P-713 XY Piezo Scanner nanopositioning stage and viewed with an inverted Olympus IX71 microscope using a 0.8NA 100× microscope air objective (Olympus LMPlanFL-100×). Light from a Hg lamp (Olympus USH-1030L) was filtered at 438 ± 12 nm for widefield fluorescence imaging, and images were recorded by a CCD camera (Photonic Science CMOS). In a confocal setup, lifetime and photon-correlation histograms were obtained using a 405 nm pulsed laser (pulse width of 100 ps) at 2.5 MHz (PicoQuant LDH series), and optical lithography was performed by a supercontinuum laser (NKT Photonics SuperK Extreme with a SuperK Varia filter) as described previously. The emitted light was spectrally filtered by a 630 ± 46 nm bandpass filter (Semrock 630/92 nm BrightLine®) and spatially filtered by a 150-μm pinhole. Time-resolved photoluminescence and time-correlated single-photon counting were performed using PicoHarp300 and two MPD PDM series single-photon avalanche diodes in a Hanbury-Brown and Twiss arrangement. The antenna emission pattern was recorded using a 0.95NA 100× microscope air objective (Olympus MPLAPON 100×) by Fourier plane imaging in the far field with an electron-multiplied CCD (EMCCD) camera (Andor iXon Ultra 897). The AFM images were recorded using a Veeco Dimension 3100 microscope with a Bruker RTESPA (Model: MPP-11120-10) tip in tapping mode. The transmission electron microscopy images were recorded with a Joel F2010 microscope.

## Supplementary information

Supplementary Information: Extreme multiexciton emission from deterministically assembled single-emitter subwavelength plasmonic patch antennas
