# Supplementary material for: Extreme multiexciton emission from deterministically assembled single-emitter subwavelength plasmonic patch antennas
Source: Light Sci Appl. 2020 Mar 4;9:33. doi: 10.1038/s41377-020-0269-0 (PMC7054275; doi:10.1038/s41377-020-0269-0)
Supplement: Supplementary file 1 — Supplementary Information: Extreme multiexciton emission from deterministically assembled single-emitter subwavelength plasmonic patch antennas [file 41377_2020_269_MOESM1_ESM.docx]

**Supplementary Information**

***Extreme multiexciton emission from deterministically assembled single-emitter subwavelength plasmonic patch antennas***

Amit Raj Dhawan^1,2^, Cherif Belacel^2,3^, Juan Uriel Esparza-Villa^2^, Michel Nasilowski^4^, Zhiming Wang^1^, Catherine Schwob^2^, Jean-Paul Hugonin^5^, Laurent Coolen^2^, Benoît Dubertret^4^, Pascale Senellart^3^, Agnès Maître^2*^

^1^Institute of Fundamental and Frontier Sciences, University of Electronic Science and Technology of China, Chengdu 610054, People’s Republic of China

^2^ Sorbonne Université, CNRS, Institut des Nanosciences de Paris, UMR 7588, 75005 Paris, France

^3^Centre de Nanosciences et de Nanotechnologies et de Nanostructures, CNRS UMR9001, Université Paris-Saclay, 10 boulevard Thomas Gobert, 91120 Marcoussis, France.

^4^Laboratoire de Physique et d'Etude des Matériaux, ESPCI-ParisTech, PSL Research University, Sorbonne Université, CNRS UMR 8213, 10 rue Vauquelin 75005 Paris, France

^5^Laboratoire Charles Fabry, Institut d’Optique Graduate School, CNRS UMR 8501, Université Paris Saclay, 2 avenue Augustin Fresnel, 91127 Palaiseau Cedex, France

***Corresponding author**. Email: [agnes.maitre@insp.upmc.fr](mailto:agnes.maitre@insp.upmc.fr). Telephone: +33 1 44 27 42 17

# S1 Quantum dot fabrication

Chemically synthesized CdSe/CdS core/shell colloidal quantum dots (QDs) were used in this work. Firstly, the CdSe cores are synthesized using a protocol based on the work of Peng *et al.* [1, 2] and then the CdS shells are grown by continuous slow injection.

## S1.1 CdSe core synthesis

In a 100 ml three-neck flask, 750 μl of 0.5 M cadmium oleate [Cd(C18H34O2)2], 1.5 ml of TOPO [trioctylphosphine oxide, [CH3(CH2)7]3PO], 1.5 ml of octadecene [C18H36] are added. The mixture is degassed at 70°C for 3 hours. Then argon gas is let in and the solution is heated from 20°C to 300°C. Very swiftly, 4 ml of trioctylphosphine selenide [TOPSe, (C8H17)3P=Se] is added, which is followed by the addition of 3 ml oleamine or oleylamine. The mixture is then annealed for 8 minutes. The flask is let to cool down and this stops the synthesis of the CdSe cores. For room temperature colloidal stability of the dots, 0.1 ml of oleic acid [C18H34O2] is added to the solution. The dots in the solution are precipitated by adding ethanol [C2H5OH]. The precipitated CdSe nanocrystals are collected and suspended in hexane (about 5 ml). The final diameter of the dots affects their photoluminescence spectrum.

## S1.2 CdS shell growth

The precipitated CdSe cores from the above step are put in a three-neck flask with 5 ml of octadecane (C18H38) and 50 mg of cadmium is added to it. The mixture is degassed at 70°C for 30 minutes and put under argon flow. Then 5 ml of 8.1 M solution of sulphur in octadecene, 1 ml of 0.5 M cadmium oleate, and 1 ml of oleylamine (C18H35NH2) are added at the injection rate of 2 ml/hour for the first 2 ml and the remaining is injected at 18 ml/hour. The mixture is heated at 300°C for 10 minutes. It is then cooled down to room temperature and the nanocrystals are precipitated with ethanol, centrifuged and dispersed in 5 ml of hexane (C6H14). Finally, we have a colloidal dispersion of CdSe/CdS core/shell nanocrystals in hexane. Figure F1 shows a schematic of a quantum dot with ligands.


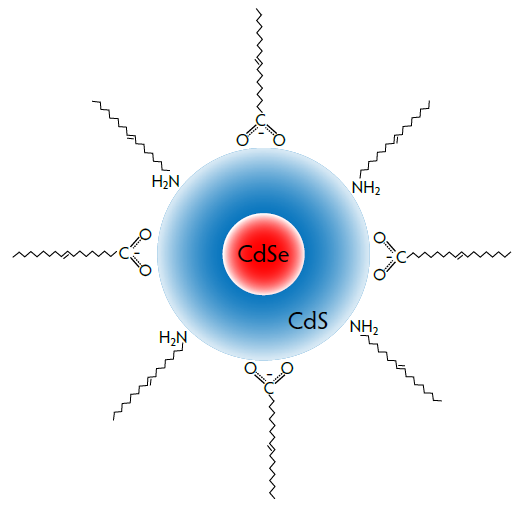


***Figure F1 | CdSe/CdS quantum dot with ligands******.*** *The ligands hold the quantum dot in the dispersion. We use hexane as the solvent but any other nonpolar solvents like toluene or chloroform can be used. The ligands attached to the shell consist of an 18-atoms carbon chain, which is terminated by either COO- or NH2. The COO- and NH2 attach to the quantum dot and the 18-atoms alkyl chain is oleophilic—this ensures the colloidal stability. The double bond between the 9th and 10th carbon atom ensures the stability of the nanocrystals in nonpolar solvents like hexane and chloroform.*

## S1.3 Optical properties of core/shell CdSe/CdS colloidal quantum dots

These QDs have a high absorption cross-section, are very bright at room temperature, and show almost no blinking (Figure F2(b)) [3]. Under UV excitation, they emit at 635 nm with a spectral width of 30 nm. Under weak pulsed excitation, they inherently emit single photons with a second-order photon cross-correlation factor *g*^(2)^(0) = 0.2–0.3, whereas under high excitation, the *g*^(2)^(0) peak rises quickly and multiple photon emission is obtained. The multiexcitons created by the absorption of exciting photons [4] relax in a series of cascaded transitions [5, 6] (Figure 4(d) of the paper), which can be either radiative or non-radiative depending on the efficacy of the radiative decay channels versus the non-radiative Auger channels. For QDs of small size, multiexciton recombination is mostly non-radiative because the radiative multiexciton decay rate is much lower than the non-radiative Auger rate, so the final exciton to ground state transition results in the emission of a single photon. As the Auger effect is inversely proportional to the QD volume [7], it is much less efficient in the case of large QDs. Therefore, because of large absorption cross-section and inefficient Auger channels, under high intensity excitation [8, 9], the probability of multiexciton increases and our “giant” QDs easily show multiphoton emission. The excited state to ground state decay of the QD by multiexciton to exciton to ground state recombination cascade leads to a time-dependent emission intensity given by *I* (*t*) = *a*_fast_*e*−*t*⁄*τ*_fast_ + *a*_slow_*e*−*t*⁄*τ*_slow_ + *c*, where *a*_fast_ and *a*_slow_ are the amplitudes corresponding to the fast and slow decay times, *τ*_fast_ and *τ*_slow_, resp., and *c* is the noise. Exciton recombination and single photon emission are associated with the slow component (*τ*_slow_ = $\tau_{\text{X}}^{\text{Antenna}}$), whereas the fast component is due to biexciton, triexciton, and other high order multiexciton recombinations [6]. Depending on the resolution of the measurement system, the coefficient(s) and decay time(s) can be arranged accordingly.

The emission decay of a typical QD (Figure F2(a)) clearly depicts two slopes, the fast decay for multiexciton recombination and the low decay rate for exciton recombination. We note an increase in the contribution of the fast mutiexciton recombination as the excitation laser intensity is increased, which is the signature of a larger multiexciton generation at higher pumping.


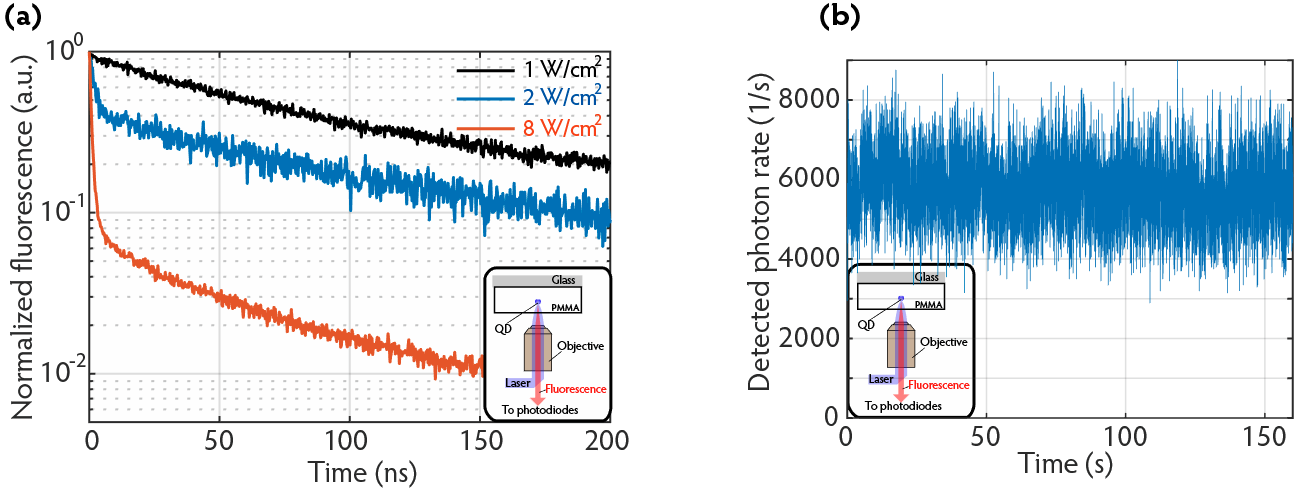


***Figure F2 | Single emitter plasmonic patch antenna operation principle and its emitter. (a)*** *The emission decay histogram of the single QD shown under laser excitation intensities of 1 W/cm^2^ (black trace), 2 W/cm^2^ (blue trace) and 8 W/cm^2^ (red trace). The rapidly falling initial components of the blue and red curves are due to multiexciton emission. Higher laser intensity on the QD leads to more multiphoton emission, which is evident in the blue and red traces but is almost absent in the mono-exponential black curve decay. The inset depicts the measurement scheme.* ***(b)*** *Non-blinking behavior exhibited in the photoluminescence time-trace (binning time of 20 ms) of a single QD for a laser (405 nm, pulse duration 100 ps, repetition rate 2.5 MHz) excitation power of 2 W/cm^2^.*

# S2 Lithography protocol for deterministic and controlled fabrication of single emitter plasmonic patch nano-antennas

The optical characterization and lithography setup is shown in Figure [F3](#_bookmark1). The photon statistics presented here were obtained by exciting the sample with a PicoQuant 405 nm pulsed laser with a repetition rate of 2.5 MHz and a pulse-width of 100 ps, and using a 0.8NA, 100x air objective from Olympus. The photon detection rate was recorded using Micro Photon Devices PDM series avalanche photodiodes and PicoHarp 300 single photon counting system. An NKT Photonics SuperK EXTREME high-power supercontinuum laser at a repetition rate of 79 MHz was used for lithography.


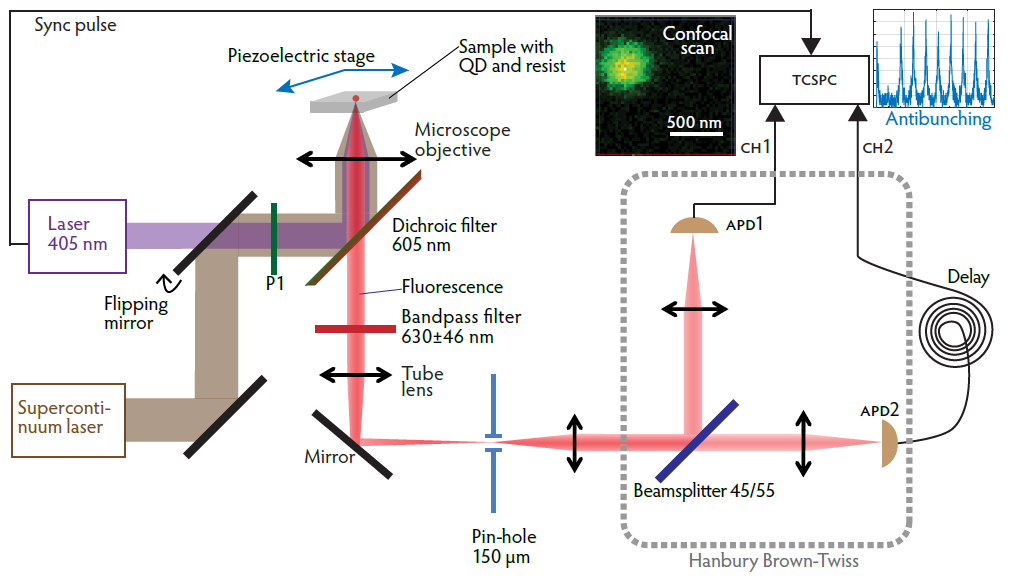


***Figure F3 | Supercontinuum laser lithography.*** *A QD is confocally scanned with a 473–478 nm laser wavelength range and then the resist in that area is ablated with a high intensity laser at 550–605 nm. Photon emission statistics are recorded under 405 nm pulsed laser excitation at 2.5 MHz. The flipping mirror decides which laser beam goes into the microscope. At position P1, we place a laser power meter and a removable mechanical shutting system. The abbreviations used here are: APD (avalanche photodiode), CH (channel), and TCSPC (time correlated single photon counting).*


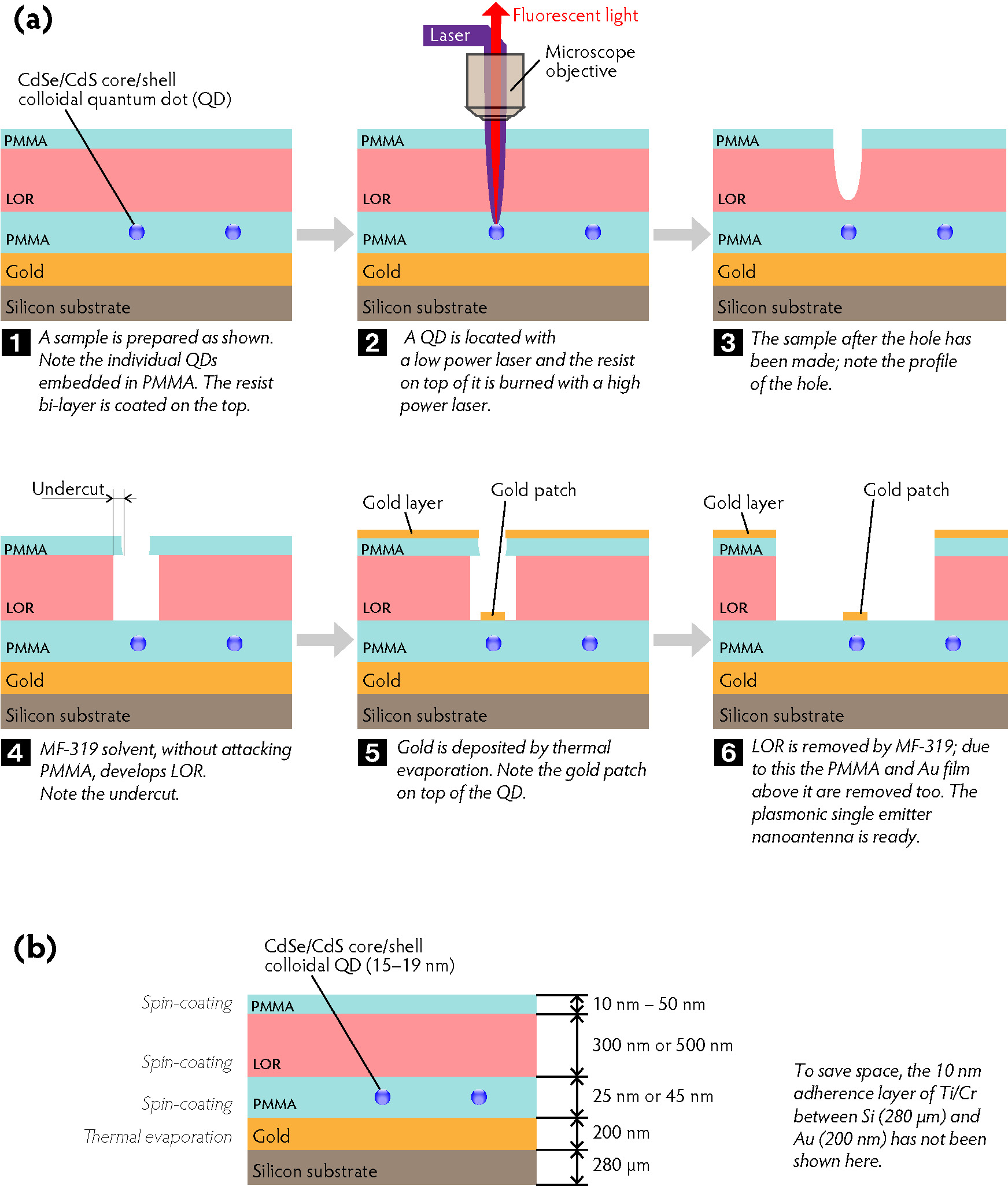


***Figure F4 | Realization of a single emitter plasmonic nano-antenna by deterministic in-situ lithography. (a)*** *illustrates the lithography protocol, and* ***(b)*** *shows the thickness of the layers and their deposition method. By adjusting the solution concentration, the spin-coated film thickness is varied.*

Deterministic lithography on fragile single emitters can be very challenging. With the motivation of performing deterministic and controlled lithography on fragile single emitters with very high precision, we devised an optical lithography protocol offering the following advantages:

- The lithography protocol works at room-temperature and in ambient conditions (e.g., atmospheric pressure). It performs lithography on fragile single emitters without damaging them. The high intensity light used in optical lithography can photodegrade or even destroy the emitter during the writing step. By selecting the appropriate range of wavelength, we can write on the resist without damaging the emitter beneath it.
- We select lithography resists with low luminescence, which allow the observation of single emitters with low fluorescence signal embedded below the resist. The high photoluminescence of a typical photoresist can make low fluorescence single emitters embedded below it invisible.
- Our technique circumvents the problem of unintended exposure of the photoresist during the localization of single emitters. Generally, photolithography requires exposing the photoresist with light of appropriate wavelength and intensity before the etching step [10]—exposure of the photoresist results in local chemical modification of the photoresist material, which is then selectively treated. Locating a single emitter (which has considerably lower fluorescence) at room-temperature requires scanning an area with light intensity which is high enough to cause unintended exposure of the photoresist in the entire scanned area, thus making any further localized exposure or lithography impossible. We resolved this issue by using another kind of optical resist, which is burnt during lithography and is not prone to typical chemical exposure problems of usual photoresists. Therefore, we can use moderately high intensity light for observing the emitters without being concerned with resist exposure. After locating the emitter, the laser wavelength is changed and the intensity is increased to burn the optical resist locally above it without damaging it.

Figure [F4](#_bookmark2) illustrates various steps of the protocol. On a Si wafer (280 μm), a 10 nm layer of Cr/Ti is evaporated, and then a 200 nm thick layer of Au is deposited by thermal evaporation. The Cr/Ti layer serves adheres the Au layer to the Si substrate. Then a 10 nm layer of PMMA is spin-coated on the Au substrate. An appropriately concentrated dispersion of CdSe/CdS QDs in hexane is spin-coated on it to obtain well-distributed individual QDs. A PMMA film of 35 nm thickness is spin-coated on the QDs, which results in embedding individual QDs in a 45 nm thick PMMA matrix. To perform optical lithography on the sample, a 300 nm thick lift-off resist [11] layer is spin-coated. Further, a thin layer of PMMA is spin-coated. The sample is ready for imaging after this step 1 of the protocol. At step 2, the sample is fixed on a piezoelectric nanopositioning stage and the QD position is noted by recording its fluorescence after exciting it with a low intensity focused laser. The fluorescing QD is positioned at the center of the laser spot using the piezoelectric stage, and high intensity light is used to burn the resist bi-layer without photobleaching the QD as shown in step 3. The size of the burnt hole can be controlled by varying the laser exposure time and intensity. At step 4, the sample is developed to create an undercut in the PMMA film, which permits the final lift-off at the final step. Then a 20 nm thick Au layer is deposited at step 5. At step 6, the sample is lifted-off, and we obtain patch antennas of controlled size and at the intended positions.

# S3 Lithography precision

Our extensively characterized and elaborate spin-coating methods ensure 3 nm vertical precision of embedding the emitter between the gold film and the gold patch. The thickness of the PMMA films on several samples was measured by ellipsometry, where 2–3 different areas were measured on each sample. After spin-coating several samples with the same PMMA solution and spin-coating parameters, we noted reproducibility or repeatability of the process. By measuring 2–3 areas on a sample, we noted the homogeneity of the spin-coated film. In these studies, the spin-coated PMMA layer was baked as in the actual fabrication process. Through these measurements and error analysis, we find the precision of embedding the emitter between our two spin-coated and baked PMMA films of total thickness of 30–45 nm to be at least 3 nm. We found the accuracy of film thickness to be from about 5–7% of the total thickness of the film, which is 3 nm for a 45 nm film.

Table 1 displays the thickness of a PMMA film measured by ellipsometry. The nominal thickness of the film was 40 nm. The measured wavelength is 40.4±1.8 nm. The small error justifies the accuracy of the deposition methods.

***Table 1*** ***| Film thickness from ellipsometry***

| **Sample** | **Area on sample** | **Film thickness measured by ellipsometry (nm)** |
| --- | --- | --- |
| Sample A | Area 1 | 40.4 ± 0.2 |
| Sample A | Area 2 | 39.5 ± 0.3 |
| Sample A | Area 2 | 41.5 ± 0.1 |
|  |  |  |
| Sample B | Area 1 | 42.3 ± 0.5 |
| Sample B | Area 2 | 41.8 ± 0.4 |
| Sample B | Area 3 | 42.4 ± 0.3 |
|  |  |  |
| Sample C | Area 1 | 38.6 ± 0.3 |
| Sample C | Area 2 | 39.7 ± 0.4 |
| Sample C | Area 2 | 38.3 ± 0.2 |
|  |  |  |
| Sample D | Area 1 | 39.1 ± 0.5 |
| Sample D | Area 2 | 40.5 ± 0.3 |
| Sample D | Area 2 | 41.2 ± 0.4 |

The lateral precision of our lithography technique depends on the focusing of the laser and the imaging of the emitter fluorescence. We locate emitters by stage scanning confocal microscopy, where the TEM_00_ laser mode is focused onto a QD held on a laterally moving stage. As the stage scans the focused laser spot, the emitter probes the laser spot. Its emission is detected by single photon avalanche photodiodes and its map is created on a computer screen. As shown in the microphotoluminescence scan of Figure F5, the TEM_00_ laser spot has a Gaussian profile, and the emitter is positioned at the tip of this peak, which can be found with an uncertainty of 1 to 2 pixels on the computer screen. Typically, for locating the emitter, we scan an area of 1×1 μm of 50 × 50 pixels, which gives us a precision of 50 nm in positioning the emitter.


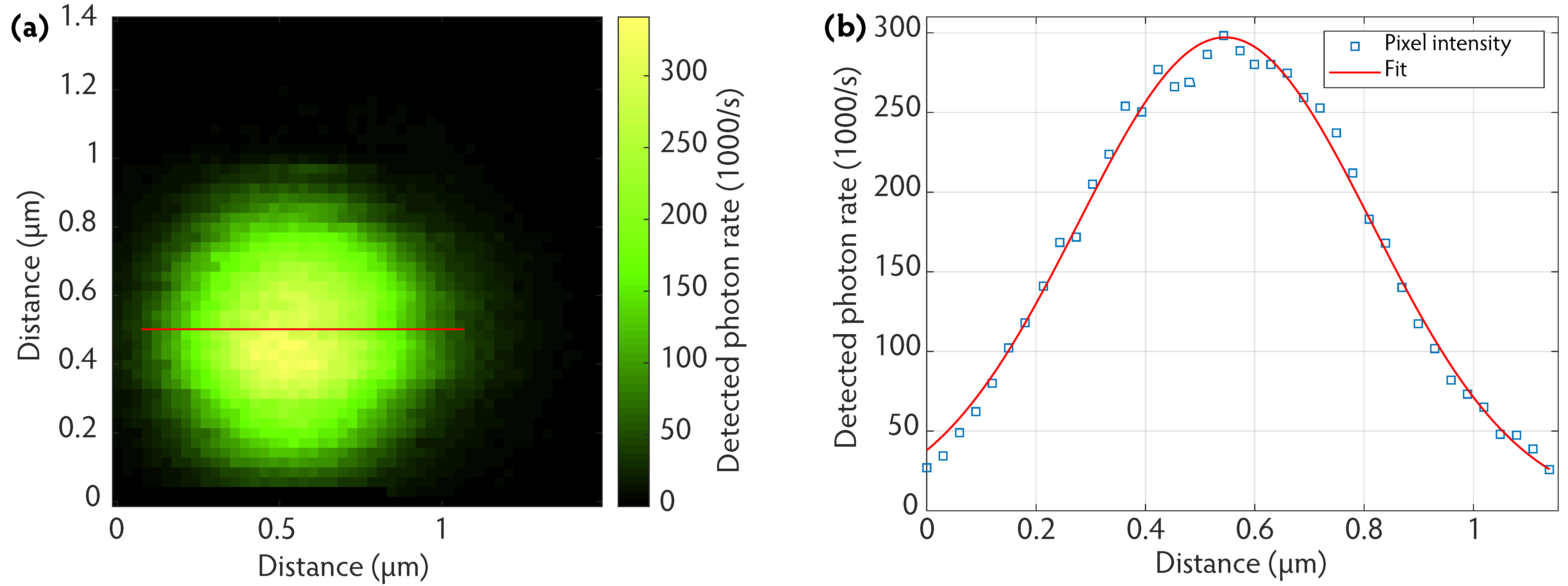


***Figure F5 | Lateral precision of locating an emitter. (a)*** *Confocal scan of a single QD excited by a TEM_00_ laser mode, and* ***(b)*** *the pixel intensity profile along its center (red line in* ***(a)****), which is fit by a Gaussian function (red). Note the pixel at the tip of the Gaussian, which signifies its center, where the emitter is located.*

The precision of the technique can be improved by incorporating more specialized strategies. For example, the lateral precision can be increased by deploying techniques like pixel reassignment [12], Airyscanning [13], and post-processing [14].

# S4 The antenna acceleration factor and the Purcell factor

In this section we will discuss the antenna acceleration factor *F*A (as defined in this paper), and the more widely known Purcell factor *F*P. The antenna acceleration factor can be calculated from the exciton emission decay rates of the QD before and after it is placed inside the antenna. Using the value of *F*A and the theory of dipole interaction near metallic surfaces, the value of the Purcell factor is found.

## S4.1 Antenna acceleration factor

We define the antenna acceleration factor *F*A as:

$F_{A}=\frac{\tau_{\text{X}}^{\text{ref}}}{\tau_{\text{X}}^{\text{Antenna}}}$ (1)

where $\tau_{\text{X}}^{\text{ref}}$ and $\tau_{\text{X}}^{\text{Antenna}}$ are the respective exciton lifetimes of the QD before and after it is placed inside the antenna. Figure [F6](#_bookmark6) (a) illustrates the measurement of *τ_x_*ref, that is the exciton lifetime of the QD placed in a PMMA layer and at a distance of 10 nm from a gold surface. Under low intensity laser excitation, our QDs (which have unity exciton quantum yield) display a monoexponential decay (black curve in Figure F2 (a)) and therefore the measured lifetime is the exciton lifetime. The emission decays of our antennas can be generally fitted with a biexponential curve:

*I* (*t*) = *a*fast*e*−*t/τ*fast + *a*slow*e*−*t/τ*slow + *c* (2)

where *I* (*t*) is the photon emission at time *t*, *a*fast and *a*slow are the amplitudes corresponding respectively to the fast and slow lifetimes, *τ*fast and *τ*slow, and *c* is the noise. For our QDs and antennas, we find that:

*τ*slow = *τ*X*.* (3)

Eq. (3) is typically valid for our QDs that have not been photo-degraded. Figure F5 shows the emission from a QD inside an antenna. The blue curve in Figure F7(a) depicts the antenna decay, which is fitted by a bi-exponential equation. The corresponding photon-correlation curve is shown in Figure F7(b) in blue color. Under weak excitation, we recorded a *g*^2^(0) of about 0.2 to 0.3 from this QD after the chemical etching step (before the deposition of gold above it). The high value of *g*^2^(0) of 0.5 signifies the increase in biexciton emission due to the plasmonic antenna, and this can be noted as the fast component of the bi-exponential blue curve of Figure F7(a). The red curve in Figure F7(a) was obtained by temporally filtering out events from the initial 4 ns by post-processing the time-tagged data collected by the single photon avalanche photodiodes. It has been fitted with a mono-exponential equation, which includes only the exciton decay. The exciton lifetime of *τ*slow = *τ*X = 8.9 ns was used in both the fitting equations. The slight variation in the amplitudes *a*slow and the goodness of the fits of the blue and red curves, resp., can be mathematical as we are comparing a less constrained bi-exponential fit with a more constrained mono-exponential fit. The main physical message here is clear when we note the decrease in *g*^2^(0) to 0.2 (red curve of Figure F7(b)) from 0.5 (blue curve). This demonstrates that the initial fast component of the antenna decay curve is due to multiexcitons (biexcitons here) and by temporally removing the multiexciton emission we obtain a higher probability of single photon emission.


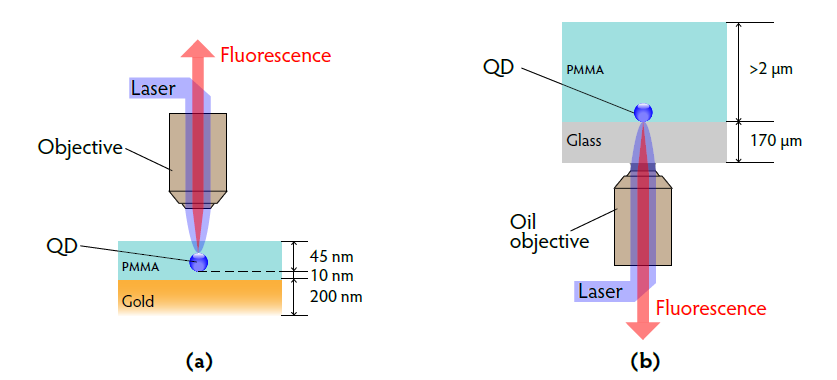


***Figure F6 | Measurement of quantum dot lifetime.*** *Figure* ***(a)*** *illustrates the setup used in this work for measurement of QD lifetime* $\tau_{\text{X}}^{\text{ref}}$*. The distance between the gold surface and the QD affects its lifetime, which can be considerably different from its lifetime in a homogeneous medium.* ***(b)*** *A schematic showing the measurement of QD lifetime* $\tau_{\text{X}}^{\text{homogeneous}}$ *in a homogeneous medium. The refractive indices of glass, PMMA, and the microscope objective oil are about 1.5.*


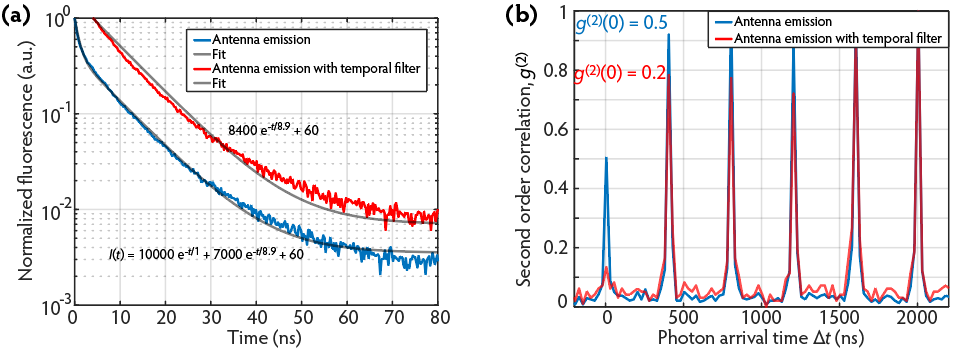


***Figure F7 | Temporal filtering of antenna emission. (a)*** *shows the emission decay of an antenna (not discussed in the paper) in blue fitted by a bi-exponential function, and by temporally filtering out the first 4 ns, the fast initial component of the blue curve is removed and the red curve (fitted by a mono-exponential) is obtained.* ***(b)*** *depicts the corresponding photon correlation curves in similar colors. Note the decrease in g*^2^(0) *as the fast multiexciton emission is temporally cut off.*

The typical exciton lifetime of our QDs (about 30–40 ns) is well-resolved by our measurement system, which has a response time of about 0.3 ns. When the same QDs are placed individually inside a plasmonic patch antenna, the emission lifetime is generally reduced. If the antenna emission is resolvable by the measurement system, it can be expressed in the form of Eq. [(2)](#_bookmark4), and the antenna acceleration factor is found using Eqs. [(3)](#_bookmark5) and [(1)](#_bookmark3).

## S4.2 Purcell factor

The acceleration of spontaneous emission of an emitter is quantified by the Purcell factor *F*P, which compares the decay rate of the emitter in a homogeneous medium with its decay rate inside another environment [15, 16] like an optical cavity or a plasmonic structure (Figure F7(a)). The Purcell factor can be expressed as:

$F_{P}=\frac{\tau_{X}^{\mathrm{homogeneous}}}{\tau_{X}^{\mathrm{Antenna}}}$ (4)

where $\tau_{\text{X}}^{\text{homogeneous}}$ and $\tau_{\text{X}}^{\text{Antenna}}$ are the lifetimes of the QD in a homogeneous medium and in the antenna, respectively. Eq. (4) can be written as:

$F_{P}=\frac{\tau_{X}^{\mathrm{homogeneous}}}{\tau_{X}^{\mathrm{Antenna}}}= \frac{\tau_{X}^{\mathrm{ref}}}{\tau_{X}^{\mathrm{Antenna}}} \times\frac{\tau_{X}^{\mathrm{homogeneous}}}{\tau_{X}^{\mathrm{ref}}}=F_{A} \times\frac{\tau_{X}^{\mathrm{homogeneous}}}{\tau_{X}^{\mathrm{ref}}}.$ (5)

The theory of emission from an emitter placed in the vicinity of gold [17, 18, 19, 20] allows us to deduce $\tau_{\text{X}}^{\text{homogeneous}}$ from the measured $\tau_{\text{X}}^{\mathrm{ref}}$, and calculate *F*P. Our CdSe/CdS colloidal QD can be modelled as a pair of degenerate orthogonal dipoles [21]. We define the orientation of the emitter by the orientation of the normal to the plane including the two orthogonal dipole vectors—this is also called the *c*-axis. Using this model, the graph of Figure [F8](#_bookmark8) shows the normalized decay rate of an emitter embedded in a PMMA layer above a gold layer as a function of its distance from a gold surface. The normalizing factor is the lifetime of the same emitter embedded in a homogeneous infinite medium of PMMA. The refractive index of PMMA is taken as 1.5, and the refractive index of the gold layer (deposited by evaporation) was found by ellipsometry (0*.*19 + *ι*3*.*38 at 635 nm). Applying this analysis to the QD in the antenna discussed in the paper, we estimate that its lifetime on the gold surface before it was placed inside the antenna is accelerated 3 times as compared to its lifetime in homogeneous PMMA. Therefore, its Purcell factor is estimated to be 3 times the measured antenna acceleration factor.


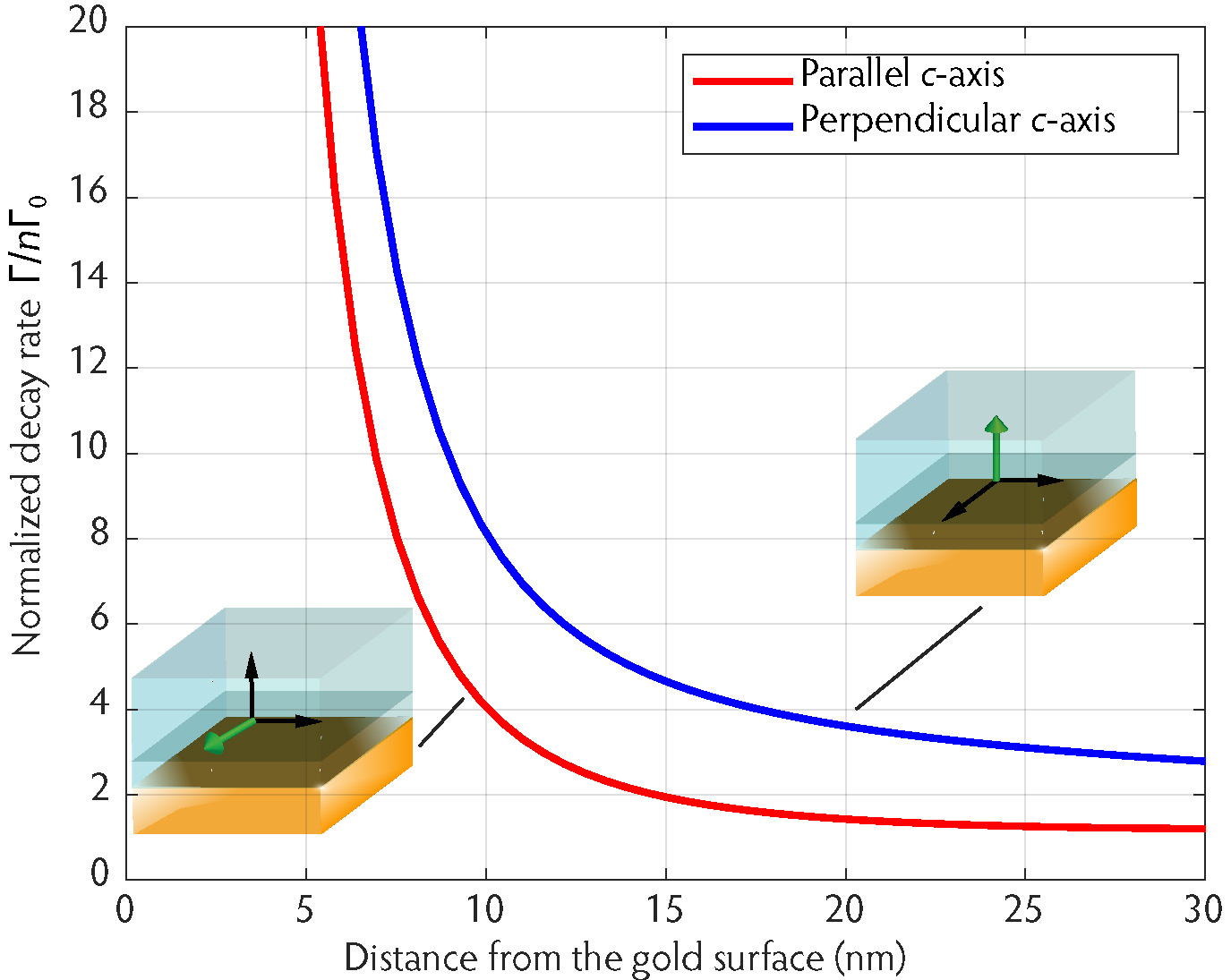


***Figure F8 | Change in decay rate of an emitter due to its proximity to a gold surface.*** *The plot shows the change in the emission decay rate of an emitter (modelled by a pair of degenerate orthogonal dipoles depicted as black arrows in the insets) as a function of its distance from a gold surface. The emitter is embedded in a homogeneous medium of PMMA. The emission decay rate increases considerably as the emitter–gold separation decreases below 50 nm. At this small separation, the emission acceleration is more when the c-axis (green arrow) is parallel to the gold surface.*

##

## S4.3 Extremely fast antenna

In the case of the fast antennas, the emission lifetime can be so short that it cannot be resolved by the measurement system. This was the case of the small and bright antenna of Figures 3, 4 and 5 of the paper, whose emission was faster than the instrument response time. Figure 4(a) of the paper shows that while measuring the decay rate of this antenna, we were just recording the instrument response function. Figure F9 plots the instrument response function (IRF) of our system under similar experimental conditions as used to measure the response of the antenna in Figure 4(a). The IRF has been fitted with a bi-exponential function. Given that the antenna response was faster than the IRF, we can say that the slowest component of the antenna was at least as fast as the IRF. Using this fact, and Eqs. (1–3) of this document, we can state the exciton lifetime of the antenna:

$\tau_{X}^{\mathrm{Antenna}}<1.5$ ns. (6)

This leads to:

$F_{A}>24$ (7)

and following Eq. (4) and (5), and the discussion of S4.2, we find that

$F_{P}>72.$ (8)


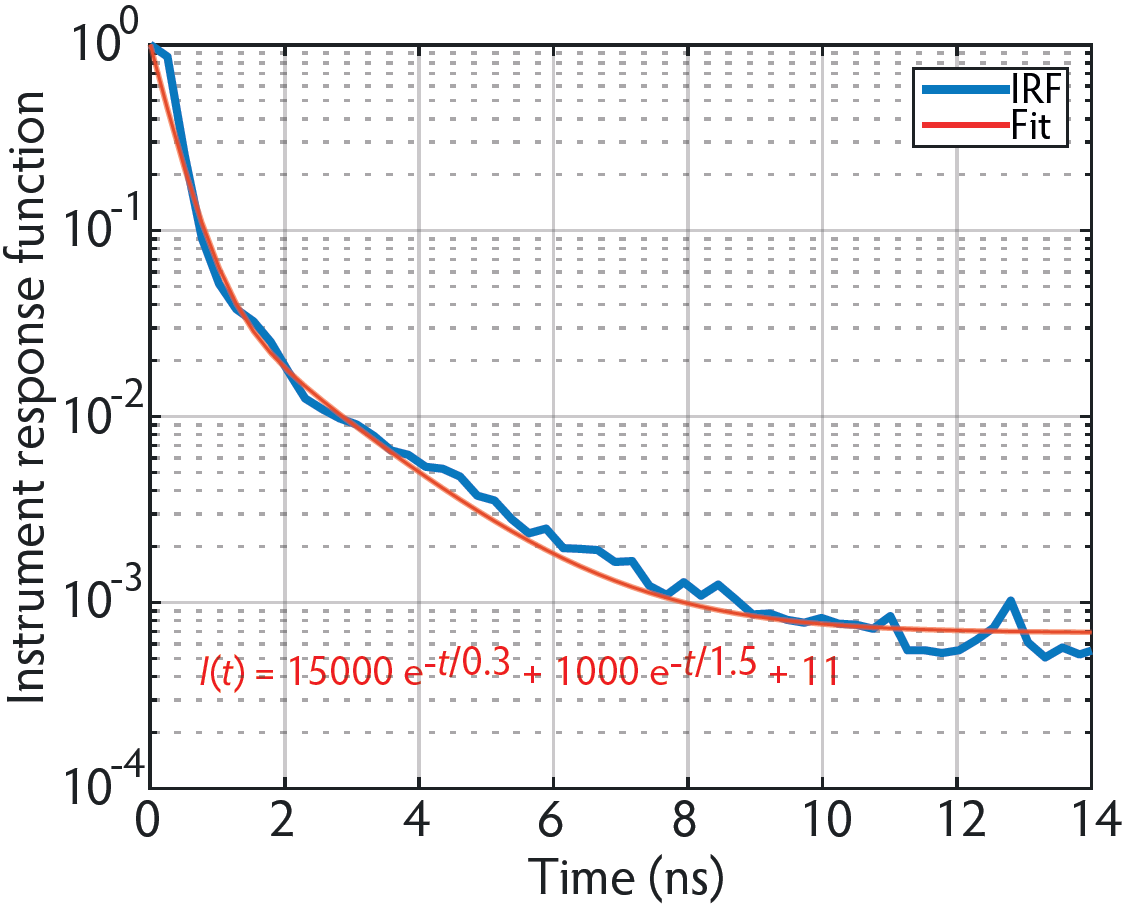


***Figure F9*** *|* ***Instrument response function with a bi-exponential fit.***

# S5 Controlled laser etching and antenna fabrication

The shape and size of the antenna mostly depends on the shape and size of the laser spot incident on the resist bi-layer. Therefore, controlling the laser leads to controlling the laser etching process. The repeatability of the laser etching process is demonstrated by the atomic force microscopy images of Figure F10, where Figures F10(a) and (b) show lattices of c holes in a resist bi-layer, and Figures F10(c) and (d) show 1.7 μm diameter holes in the same. This was achieved by changing the area of the laser spot on the sample and exposure duration. The uniformity in the size and shape of the holes in the two figures demonstrates the repeatability of the laser etching process, which directly affects the shape and size of the antenna patch.


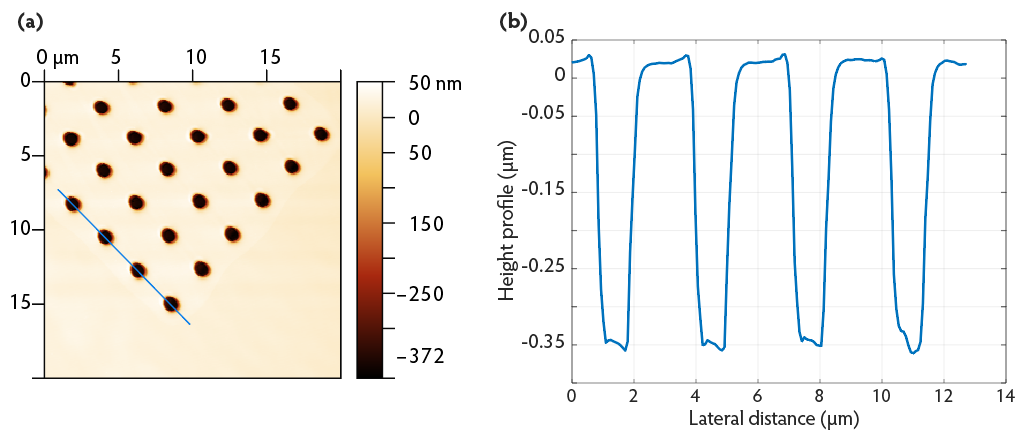

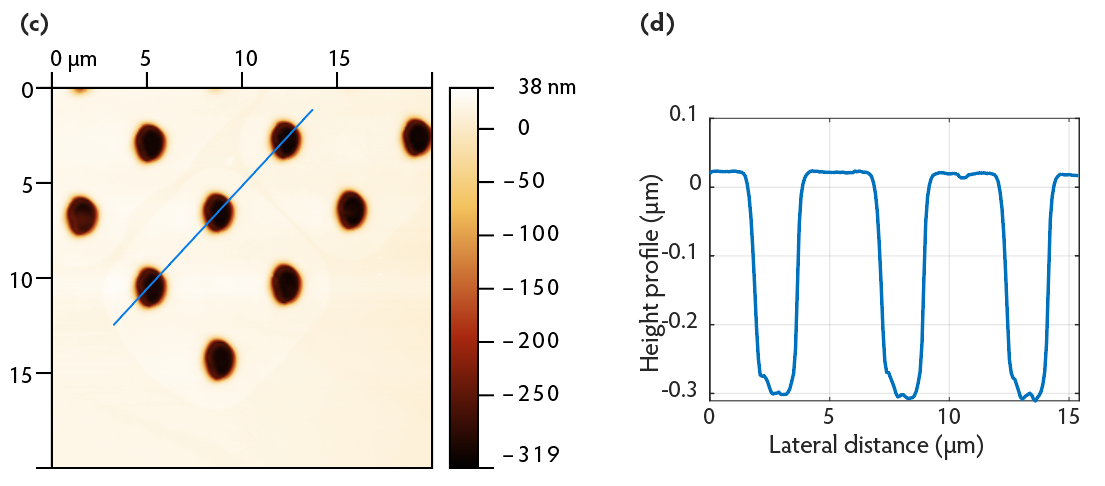


***Figure F10 | Reproducible laser etching of a resist bi-layer.*** *(a) Atomic force microscopy images showing a lattice of 1 μm diameter holes and the height profile along the indicated direction. (b) The same for 1.7 μm diameter holes. Note the consistency in shape of the holes in each lattice.*

The bright and dark field optical microscopy images of Figure F11 show three antenna patches (dots inside squares) that were fabricated using the protocol detailed here. The 15 μm squares were made to facilitate the liftoff. The scanning electron images of Figure F12 show an antenna patch from this sample. The circular shape of the patch is due to the laser spot shape.


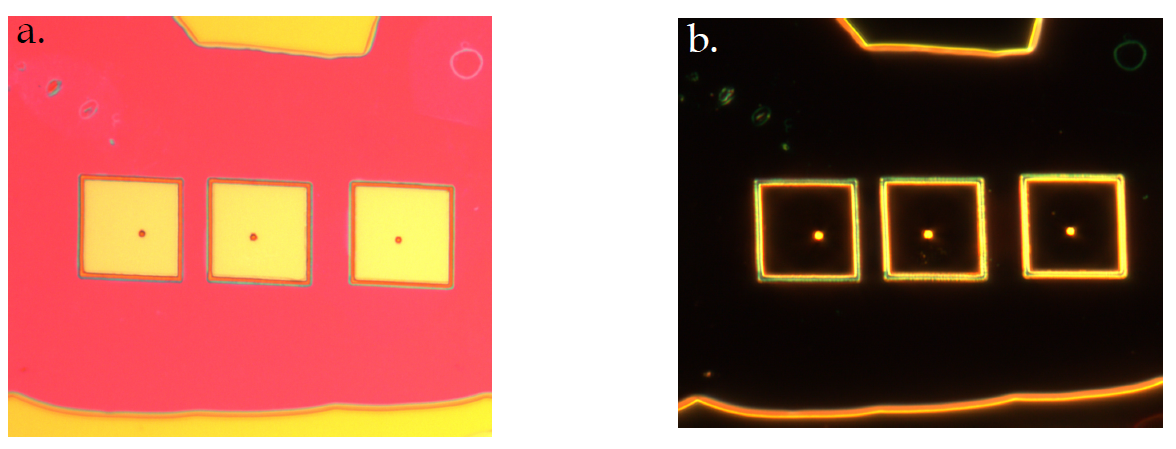


***F11 | Optical microscopy images of antenna patches. (a)*** *Brightfield microscopy images in true color of three antenna patches (red dots inside yellow squares).* ***(b)*** *Darkfield microscopy images in true color of three antenna patches (yellow dots in black squares marked by yellow boundaries).*


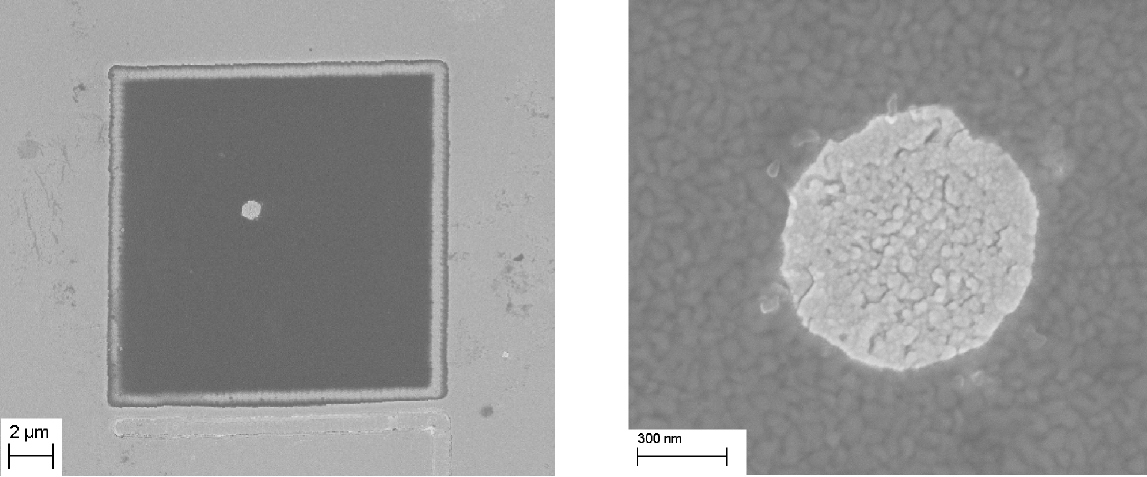


***F12 | Scanning electron image of an antenna patch.*** *The image on the left shows and antenna patch inside a square, which was magnified to obtain the image on the right. The antenna had a diameter of 0.85 μm.*

The technique can be used to fabricate antennas of different sizes, and the shape can be varied slightly as well by changing the shape of the focused laser beam. Figures 13 (a) – (d) are scanning electron microscopy images of some of the antenna patches with different sizes.

*
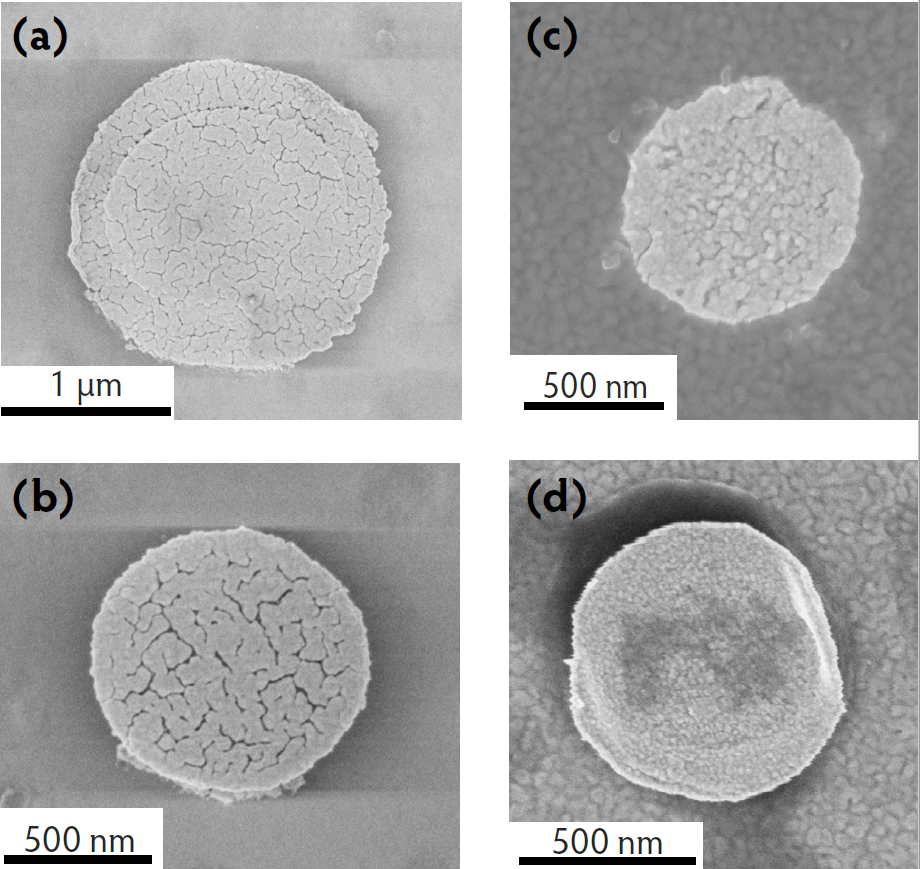
*

***F13 | Fabricated structures and optical characteristics.*** *(a) – (d) Scanning electron microscopy images of 20 nm thick Au antenna patches.*

# S6 Emission of antennas

Figure F14 shows the decay statistics of nine single emitter antennas. The dotted decay (trace 5) curve is the response of the antenna discussed in the paper—due to its very fast decay, we were recording instrument response function. Antennas 1 – 3 were accelerated but slow enough to be measured by the system. Due to the rapidity of antenna 4 (overlapping with trace 5), we recorded the instrument response function.

As plasmonic systems can be highly accelerated, while recording emission characteristics of these plasmonic single emitter antennas, we measure the instrument response function. The figure depicts even faster decays than the one discussed in the paper for four other antennas (traces 6, 7, 8, and 9), which were measured on systems with a faster instrument response. However, the antennas were so quick that we again measured instrument response functions.

*
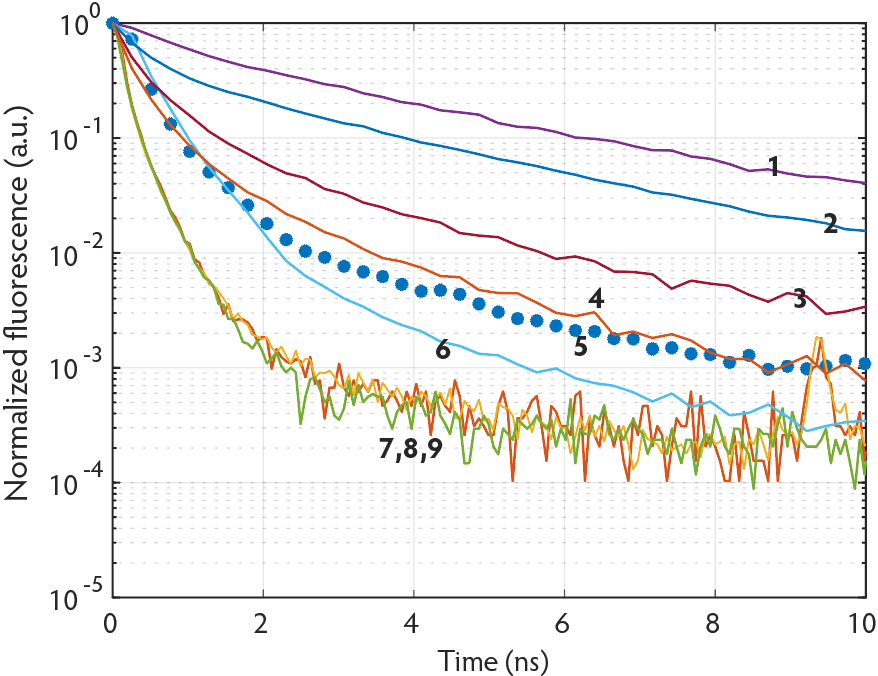
*

***F14 | Emission decay statistics of several single emitter antennas.*** *The dotted curve in blue is the antenna discussed in the paper.*

As explained in the paper, due to the acceleration of emission, the radiative decay rate can overcome the non-radiative decay rate in our antennas, which makes them significantly brighter. Another example of this is seen in Figure F15, which shows the emission characteristics of the antenna 4 of Figure F14. Figure F15(a) is a widefield fluorescence image this bright antenna, where the fluorescence of the QDs outside antennas could not be recorded sufficiently. The high directionality of this antenna, which contributes to efficient photon collection, is illustrated by the far-field Fourier plane measurement of its radiation pattern (Figures F15 (b) and (c)).


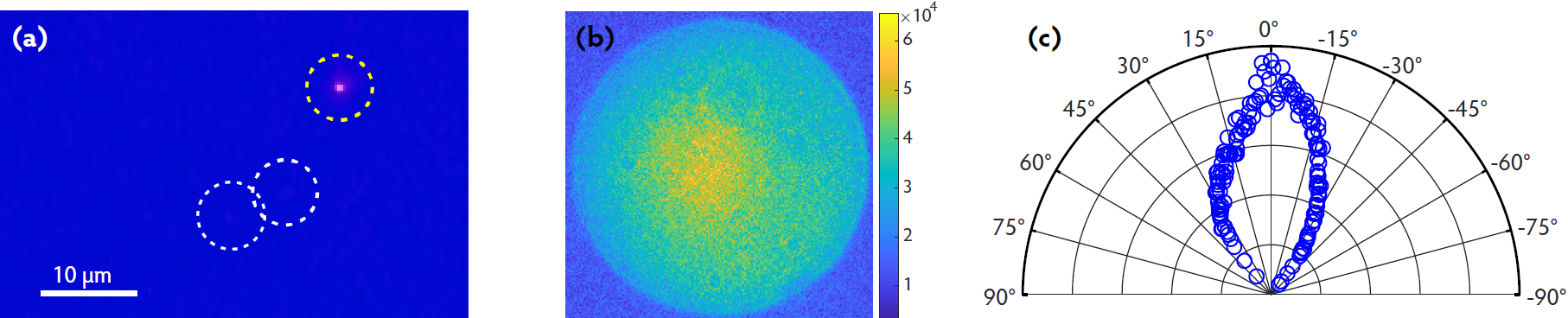


***F15 | Fabricated structures and optical characteristics.*** *(a) Brightfield fluorescence image (mercury lamp excitation filtered at 438±12 nm) of an antenna (inside the yellow dashed circle) and two other QDs outside antenna (white circles). (b) Emission pattern of this antenna, and its polar plot (c).*

## S7 Quantum dot and antenna emission spectra

After placing the QD in the antenna discussed in the paper, we observed a drastic change in its emission spectrum, which was broadened due to both blue and red shift. Note that in our observation, this is not typical of all antennas. Though the expected interaction regime in these antennas is the weak coupling, a recent study [22] has evidenced the possibility of strong coupling as well. At this stage, we are only able to display our experimental result and leave the remaining to further investigation. It can be said that the broadening of the emission spectrum is due to multiexciton emission. We have observed in our experiments and it has been documented [9] that multiexciton emission is broad with a blue-shift character. Though the blue-shifted broadening multiexciton can be attributed to multiexcitons, the red-shifted broadening has to be addressed. A 493 nm longpass filter was used to filter the emission in this experiment, which could pass all the fluorescence emission. Therefore, the sharp fall of the antenna emission between 580 nm to 550 nm is due to the nature of the emission.


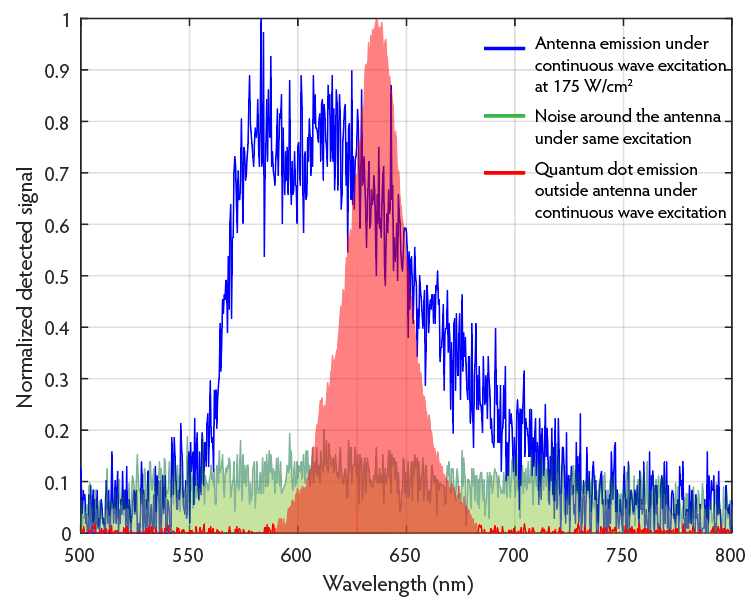


***F16 | Antenna and quantum dot normalized emission spectra.*** *The curve in red shows the emission spectrum of our typical QD under continuous wave laser excitation, which has a linewidth of about 25 nm at room-temperature. The antenna discussed in the paper showed blue-shifted and red-shifted broadening of emission. The curve in green shows the noise around the antenna under similar excitation conditions.*

## S8 Detection efficiency of the optical setup


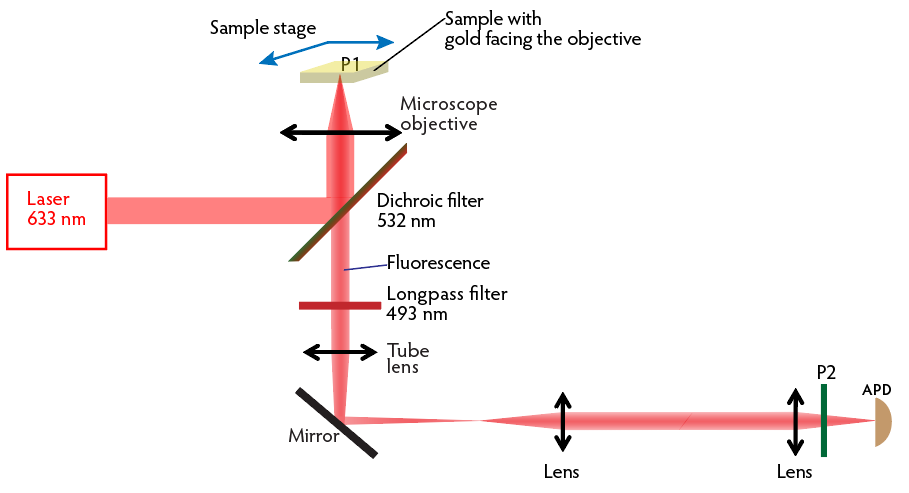


***F17 | Measurement of detection efficiency of the optical setup.***

We find the transmittance of our optical setup in the following way. A red laser is sent into the objective (0.8NA Olympus LMPlanFL-100x) and its power is measured at position P1 using a Thorlabs PM100D meter. The laser is focused on a gold sample, whose reflectivity at 633 nm is known by measurement. The reflected laser light is collected by the objective and it passes through the 532 nm dichroic and the following 493 nm filter, and lenses assembly. We then measure the 633 nm light at position P2.

P_P1_ R_gold_ T_system_ = P_P2_

Where P_P1_ and P_P2_ is the power at positions P1 and P2, resp. R_gold_ is the reflectance of gold (light of power P_P1_ is reflected by gold and passes through the objective), and T_system_ is the transmittance of the described system which includes the objective, dichroic filter, longpass filter, tube lens, mirror, and two lenses till the photodetector (APD). We note that:

P_P1_ = 0.56 ± 0.05 mW

R_gold_  = 0.92 ± 0.01

P_P2_ = 0.32 ± 0.05 mW

This gives us T_system_ =0.62 ± 0.11

The detection efficiency of the photodetector T_detector_ was not measured specifically, and we use the detection efficiency provided by the manufacturer, which is 40% or 0.4 at 630 nm.

From this we have the total detection efficiency of our system T_total_ = T_system_ × T_detector_ × 100 %

from the above values, we find **T_total_  = 0.25 ± 0.04 or 25±4 %**.

## References

## [1] X. Peng, J. Wickham, and A. P. Alivisatos, “Kinetics of II-VI and III-V Colloidal Semiconductor Nanocrystal Growth: “Focusing” of Size Distributions,” *Journal of the American Chemical Society*, vol. 120, no. 21, pp. 5343–5344, Jun. 1998. [Online]. Available: http://­dx.doi.org/­10.1021/­ja9805425

## [2] X. Peng, L. Manna, W. Yang, J. Wickham, E. Scher, A. Kadavanich, and A. P. Alivisatos, “Shape control of CdSe nanocrystals,” *Nature*, vol. 404, no. 6773, pp. 59–61, Mar. 2000. [Online]. Available: http://­dx.doi.org/­10.1038/­35003535

## [3] B. Mahler, P. Spinicelli, S. Buil, X. Quelin, J.-P. Hermier, and B. Dubertret, “Towards non-blinking colloidal quantum dots,” *Nature Materials*, vol. 7, no. 8, pp. 659–664, Jun. 2008. [Online]. Available: http://­dx.doi.org/­10.1038/­nmat2222

## [4] V. Klimov, *Nanocrystal Quantum Dots*, 2nd ed. CRC Press, 2010.

## [5] E. Moreau, I. Robert, L. Manin, V. Thierry-Mieg, J. M. Gérard, and I. Abram, “Quantum cascade of photons in semiconductor quantum dots,” *Phys. Rev. Lett.*, vol. 87, p. 183601, Oct 2001. [Online]. Available: http://­link.aps.org/­doi/­10.1103/­PhysRevLett.87.183601

## [6] G. Nair, J. Zhao, and M. G. Bawendi, “Biexciton quantum yield of single semiconductor nanocrystals from photon statistics,” *Nano Letters*, vol. 11, no. 3, pp. 1136–1140, 2011, pMID: 21288042. [Online]. Available: http://­dx.doi.org/­10.1021/­nl104054t

## [7] V. I. Klimov, A. A. Mikhailovsky, D. W. McBranch, C. A. Leatherdale, and M. G. Bawendi, “Quantization of multiparticle Auger rates in semiconductor quantum dots,” *Science*, vol. 287, no. 5455, pp. 1011–1013, 2000. [Online]. Available: http://­www.sciencemag.org/­content/­287/­5455/­1011.full

## [8] A. Rogach, *Semiconductor Nanocrystal Quantum Dots: Synthesis, Assembly, Spectroscopy and Applications*, ser. Springer ebook collection / Chemistry and Materials Science 2005-2008. Springer Vienna, 2008.

## [9] B. Fisher, J. M. Caruge, D. Zehnder, and M. Bawendi, “Room-temperature ordered photon emission from multiexciton states in single CdSe core-shell nanocrystals,” *Phys. Rev. Lett.*, vol. 94, p. 087403, Mar 2005. [Online]. Available: http://­link.aps.org/­doi/­10.1103/­PhysRevLett.94.087403

## [10] H. J. Levinson, *Principles of lithography*, 3rd ed. SPIE press, 2010.

## [11] MicroChem Corp., “LOR lift-off resists,” PDF, 2001. [Online]. Available: https://­www.nanofab.utah.edu/­svn/­public/­documents/­Non%20SOPs/­Photoresist/­LOR%20resist%20spec%20sheet2.pdf

## [12] C. J. R. Sheppard, S. B. Mehta, and R. Heintzmann, “Superresolution by image scanning microscopy using pixel reassignment,” *Opt. Lett.*, vol. 38, no. 15, pp. 2889–2892, Aug 2013. [Online]. Available: http://­ol.osa.org/­abstract.cfm?URI=ol-38-15-2889

## [13] J. Huff, “The Airyscan detector from zeiss: confocal imaging with improved signal-to-noise ratio and super-resolution,” *Nature methods*, vol. 12, no. 12, 2015.

## [14] J. McGregor, C. Mitchell, and N. Hartell, “Post-processing strategies in image scanning microscopy,” *Methods*, vol. 88, pp. 28 – 36, 2015, super-resolution Light Microscopy. [Online]. Available: http://­www.sciencedirect.com/­science/­article/­pii/­S1046202315001930

## [15] G. C. des Francs, J. Barthes, A. Bouhelier, J. Weeber, A. Dereux, A. Cuche, and C. Girard, “Plasmonic Purcell factor and coupling efficiency to surface plasmons. implications for addressing and controlling optical nanosources,” *Journal of Optics*, vol. 18, no. 9, p. 094005, 2016.

## [16] C. Belacel, B. Habert, F. Bigourdan, F. Marquier, J.-P. Hugonin, S. M. de Vasconcellos, X. Lafosse, L. Coolen, C. Schwob, C. Javaux, B. Dubertret, J.-J. Greffet, P. Senellart, and A. Maitre, “Controlling spontaneous emission with plasmonic optical patch antennas,” *Nano Letters*, vol. 13, no. 4, pp. 1516–1521, 2013, pMID: 23461679. [Online]. Available: http://­dx.doi.org/­10.1021/­nl3046602

## [17] C. Vion, P. Spinicelli, L. Coolen, C. Schwob, J.-M. Frigerio, J.-P. Hermier, and A. Maître, “Controlled modification of single colloidal CdSe/ZnS nanocrystal fluorescence through interactions with a gold surface,” *Opt. Express*, vol. 18, no. 7, pp. 7440–7455, Mar 2010. [Online]. Available: http://­www.opticsexpress.org/­abstract.cfm?URI=oe-18-7-7440

## [18] R. Chance, A. Prock, and R. Silbey, “Comments on the classical theory of energy transfer,” *The Journal of Chemical Physics*, vol. 62, no. 6, pp. 2245–2253, 1975.

## [19] L. Novotny and B. Hecht, *Principles of Nano-Optics*, 2nd ed. Cambridge University Press, 2012.

## [20] W. Lukosz, “Theory of optical-environment-dependent spontaneous-emission rates for emitters in thin layers,” *Phys. Rev. B*, vol. 22, pp. 3030–3038, Sep 1980. [Online]. Available: https://­link.aps.org/­doi/­10.1103/­PhysRevB.22.3030

## [21] C. Lethiec, J. Laverdant, H. Vallon, C. Javaux, B. Dubertret, J.-M. Frigerio, C. Schwob, L. Coolen, and A. Maître, “Measurement of three-dimensional dipole orientation of a single fluorescent nanoemitter by emission polarization analysis,” *Physical Review X*, vol. 4, no. 2, p. 021037, 2014.

## [22] H. Leng, B. Szychowski, M.-C. Daniel, and M. Pelton, “Strong coupling and induced transparency at room temperature with single quantum dots and gap plasmons,” *Nature communications*, vol. 9, no. 1, p. 4012, 2018.
